# Effects of High Temperature on Creep Behaviour of Glazed Hollow Bead Insulation Concrete

**DOI:** 10.3390/ma13173658

**Published:** 2020-08-19

**Authors:** Yu-shan Liu, Jian-yong Pang, Wei-jing Yao

**Affiliations:** 1 School of Civil Engineering and Architecture, Anhui University of Science and Technology, Huainan 232001, China; liuyushan1997@126.com (Y.-s.L.); yaoweijing0713@163.com (W.-j.Y.); 2 Engineering Research Centre of Underground Mine Construction, Ministry of Education, Huainan 232001, China; 3 State Key Laboratory of Mining Response and Disaster Prevention and Control in Deep Mine, Anhui University of Science and Technology, Huainan 232001, China

**Keywords:** glazed hollow bead insulation concrete, high temperature, creep behaviour, failure analysis

## Abstract

Glazed hollow bead insulation concrete (GHBC) presents a promising application prospect in terms of its light weight and superior fire resistance. However, only a few studies have focused on the creep behaviour of GHBC exposed to high temperatures. Therefore, in this study, the mechanism of high temperature on GHBC is analysed through a series of tests on uniaxial compression and multistage creep of GHBC, exposed from room temperature up to 800 °C. The results show a decrease in the weight and compressive strength of GHBC as the temperature rises. After 800 °C, the loss of weight and strength reach to 9.67% and 69.84%, respectively. The creep strain and creep rate increase, with a higher target temperature and higher stress level, while the transient deformation modulus, the creep failure threshold stress, and creep duration are reduced significantly. Furthermore, the creep of GHBC exhibits a considerable increase above 600 °C and the creep under the same loading ratio at 600 °C increases by 74.19% compared to the creep at room temperature. Indeed, the higher the temperature, the more sensitive the stress is to the creep. Based on our findings, the Burgers model agrees well with the creep test data at the primary creep and steady-state creep stages, providing a useful reference for the fire resistance design calculation of the GHBC structures.

## 1. Introduction

Owing to its relatively lighter weight, lower thermal conductivity, and better fire resistance compared to other types of concrete, glazed hollow bead insulation concrete (GHBC) has attracted increasing attention in recent years [1]. The glazed hollow bead is (GHB) a kind of inorganic glass mineral material with an irregular sphere made of volcanic rock and turpentine crushed into ore sand and then processed using a special puffing procedure [2]. It is characterised by stable physical and chemical properties with a vitrified and sealed surface and internal cavity [3]. The unique internal cavity and porous structure of GHB are supposed to be key in providing the characteristics of light weight, heat insulation, fire resistance, and aging resistance [4]. It is widely used in buildings such as walls, beams, and column members [5], which can significantly reduce the structural weight, foundation load, and basic engineering quantity and achieve remarkable economic benefits [6]. It not only simplifies the design of building insulation projects, but also it reduces self-weight, avoiding the weak combination of the main structure and insulation layer in numerous insulation projects [7,8]. Furthermore, it can be used in coal mine roadways to reduce thermal conductivity while meeting the strength requirements to solve the problem of heat damage [9]. So far, considerable research efforts have been devoted to understanding the mechanical properties of glazed hollow bead insulation concrete. Liu et al. [10] found out that the addition of glazed hollow bead particles in concrete could result in achieving a good balance between mechanical and thermal insulation performances due to its self-insulation property. Zhao et al. [11] elucidated the effect of the glazed hollow bead material on thermal conductivity and compressive strength, verifying the feasibility of blending glazed hollow beads into thermal insulation concrete.

With recent advances in the urbanization and increasing population density, it is noteworthy that fire has tremendous consequences for the service life of buildings and the security of life and property [12,13]. Concrete with glazed hollow bead (GHB) thermal insulation particles is a new type of green concrete that has been recently used in construction projects [14]. A great deal of research has so far focused on its high-temperature characteristics. Yao et al. [15] explored the apparent phenomena and mechanical properties of GHBC from room temperature to 1000 °C. Based on the findings from their study, the dehydration phenomenon at high temperatures modifies the microstructure and decreases the strength. Chen et al. [16] concluded that the addition of GHB can increase the porosity while obviously reducing the damage to the concrete at high temperatures.

Concrete exhibits time-dependent deformation under long-term loads; that is, creep behaviour. The creep behaviour of concrete affects its safety and durability, especially in massive concrete engineering [17]. The compression members such as columns and beams in building structures and the lining of roadways are often exposed to high stress. Furthermore, these structures may catch fire and temperature will increase to the accidental condition. Furthermore, the short-term creep for 3 h of fire exposure is up to 32 times greater than that of one-year ambient temperature creep [18]. Therefore, the creep behaviour of concrete at high temperatures is critical for designing fireproof structures. Moreover, the creep of concrete increases considerably above 500 °C. Indeed, the creep behaviour is mainly influenced by the aggregate type, stress level, loading age, and temperature [19]. The influence of polypropylene fibre on the creep behaviour of high-strength concrete after high temperatures was discussed by Wu et al. [20]. Tao et al. [21] studied the transient strain of self-compacting concrete under variations of temperature level, heating rate, and stress level. Previous studies indicated that the creep behaviour of concrete depends on the aggregate type and temperature [22,23]. The interface zone of GHBC, exposed to high temperature, shows differences from other types of concrete owing to the unique microstructure with the GHB embedded within the cement matrix [15]. However, little is known about the creep behaviour of GHBC exposed to high temperatures.

Therefore, this study aims to: (1) investigate the loss of weight and compressive strength of GHBC after exposure to high temperature; (2) explore the mechanism of high temperature on GHBC from both the failure mode and microstructure change; and (3) further study the creep behaviour of GHBC, exposed to high temperature, by conducting multistage creep experiments on GHBC subjected to temperatures of up to 800 °C. This study has potential applications in supporting the fire resistance design calculation of GHBC structures.

## 2. Materials and Methods

### 2.1. Purpose and Scope

The main purpose of this study is to investigate the mechanical properties and creep behaviour of GHBC after exposure to high temperature, which will be useful in the fire safety designs of GHBC structures. The compression tests and multistage creep tests of GHBC, exposed from room temperature up to 800 °C, were carried out. The microstructure of concrete was observed using a scanning electron microscope (SEM) (Hitachi S-3400N, Tokyo, Japan). The primary creep and steady-state creep are analyzed using the Burgers model.

### 2.2. Raw Materials

The binder material used in the experiment consists of the Chinese standard Portland cement and fly ash. The Portland cement has 29.99 MPa and 42.5 MPa compressive strength when aged 3 and 28 days, respectively. The chemical composition of binder material is shown in Table 1. A continuous grading of crushed limestone with particle sizes of 5–10 mm and an apparent density of 2780 kg/m^3^ is used as the coarse aggregate. River sand with a fineness modulus of 2.8 is selected as the fine aggregate. A high-performance water reducer (HPWR) with a water-reducing rate of 30% is employed to guarantee fluidity and water retention.

**Figure 1:**
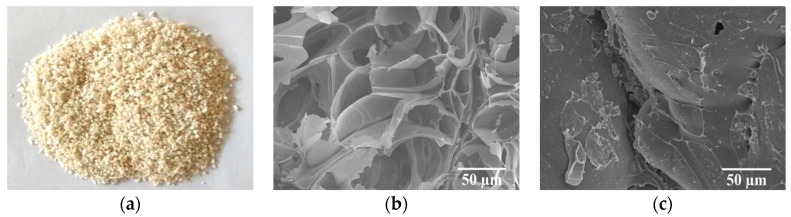
The appearance and micro-structure of the glazed hollow bead particles: (**a**) appearance; (**b**) the core; (**c**) the shell.

Table 2 represents the physical properties of GHB, used for preparing the concrete. The appearance and microstructure of GHB are presented in Figure 1a–c.

### 2.3. Mix Design

The mix proportions of GHBC, which conform to the specifications for the mix proportion design of ordinary concrete [24], are presented in Table 3. To prevent excessive water absorption of light aggregate in the mixing process from affecting the workability of concrete, the glazed hollow beads are immersed in water for 2 h until reaching saturation before mixing.

### 2.4. Experimental Design

#### 2.4.1. Sample Preparation

The dimensions of the cylinder specimens are Φ50 × 100 mm. The specimens are placed in the laboratory conditions at 20 ± 2 °C and 70% relative humidity, followed by removal of the cylinders from the moulds the next day and curing them in saturated Ca(OH)_2_ solution at 20 ± 2 °C for 56 d.

After that, all the specimens are placed in an oven at a temperature of 105 ± 5 °C for 24 h prior to achieving the target temperature to prevent bursting during heating from excessive moisture content [15]. The specimens are then heated to the target temperatures of 200 °C, 400 °C, 600 °C, and 800 °C, at a rate of 5 °C /min using a box-type resistance furnace. After rising to the target temperature, the temperature is kept constant for 3 h to ensure that the interior temperature of the specimen is the same as the furnace. Subsequently, after turning off the power, the furnace is allowed to cool about 100 °C. Afterwards, the samples continue to cool to room temperature. The dry specimens are then preserved in sealed polyethylene bags until the day of testing. In addition, specimens at room temperature are set as the control group.

#### 2.4.2. Test and Characterization

Using a CLY15016 electronic creep machine (SINOTEST, Changchun, China), the uniaxial compression and creep tests of the GHBC specimens after exposure to different high temperatures are carried out. The uniaxial compression test is carried out at a rate of 0.1 mm/min until the specimen fails to obtain the compressive strength, recorded as *f_c_*.

**Figure 2:**
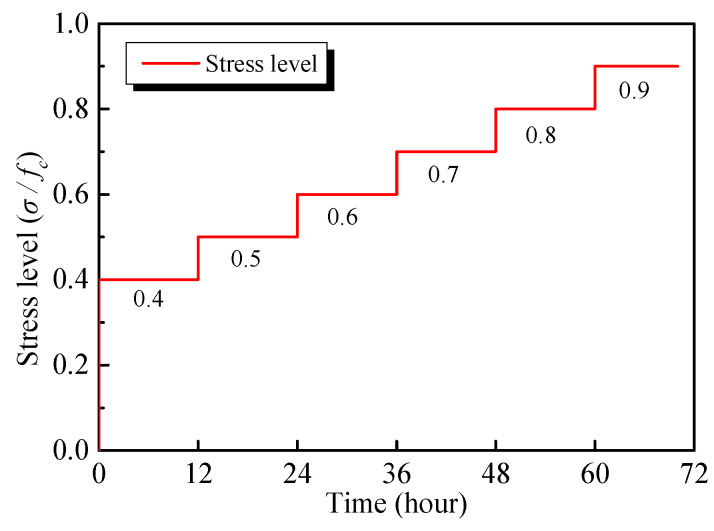
The time-stress level curve for the multistage compression creep.

**Figure 3:**
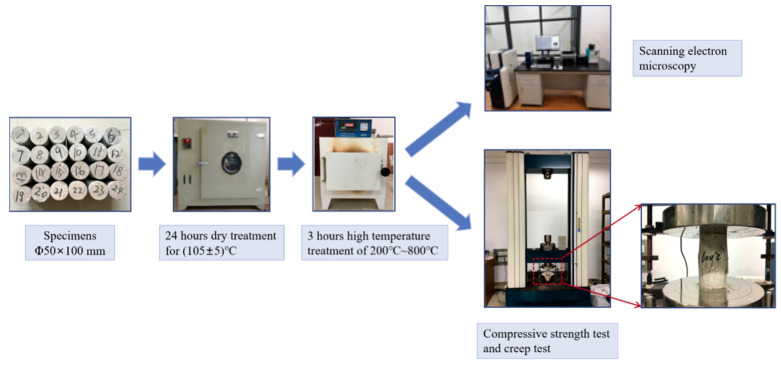
The test procedure.

In the multistage creep tests, the stress is incrementally applied step-by-step. A small preload of 0.25 MPa is applied to the specimen before the test to ensure the specimen and test device are aligned and centred properly. The stress at the first stage is 40% of the compressive strength, which is recorded as *σ* = 0.4*f_c_*. Afterwards, each stage is increased by 10% of the compressive strength, and the stress of each stage is maintained for 12 h. The time-stress level curve is shown in Figure 2. The load rate is set at 0.5 MPa/s. Data is automatically collected during the experiment, with a sampling interval of 5 s during the loading and 10 min during the stabilized period. The detailed test procedure is illustrated in Figure 3.

Subsequently, a microstructure observation between the glazed hollow beads and the cement matrix is performed using a Japanese Hitachi S-3400N scanning electron microscope.

## 3. Results and Discussion

### 3.1. Mechanical Properties

#### 3.1.1. The Loss of Weight and Compressive Strength

**Figure 4:**
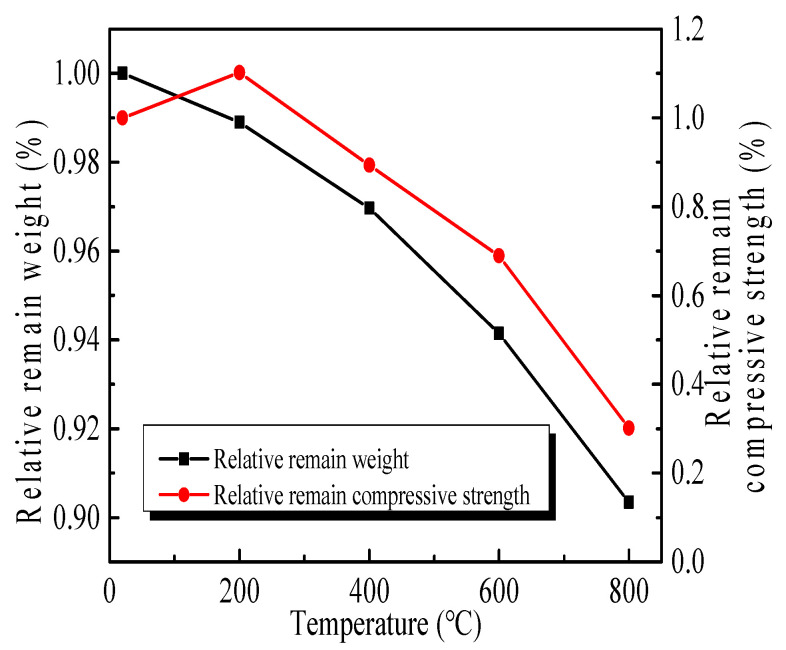
The loss of weight and compressive strength of GHBC exposed to high temperatures.

The influence of high temperatures on the weight and compressive strength of GHBC is demonstrated in Figure 4, displayed as specific values with respect to that of GHBC at room temperature. The compressive strength of GHBC decreases with the increasing temperature, except for the specimens exposed to 200 °C, and the density decreases accordingly. This is consistent with previous observations reported by other researchers [25,26].

The results reveal that the weight loss is less than 2%, primarily due to free water evaporation below 200 °C. However, the compressive strength reaches a maximum of 33.63 MPa at 200 °C, suggesting an increase of 10.17% compared to the reference GHBC at room temperature with a compressive strength of 30.52 MPa (Figure 4). The reason for the increased strength may be attributed to the secondary hydration of cementitious binder during high-temperature curing, which makes the paste denser [27].

**Figure 5:**
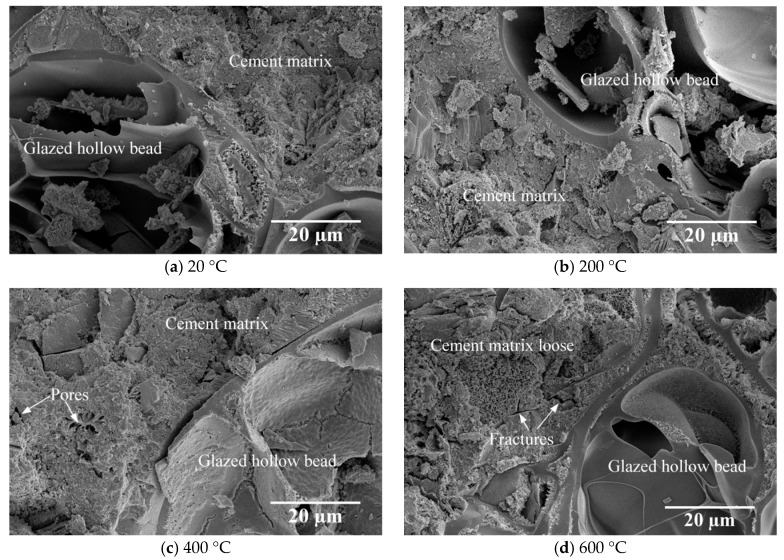

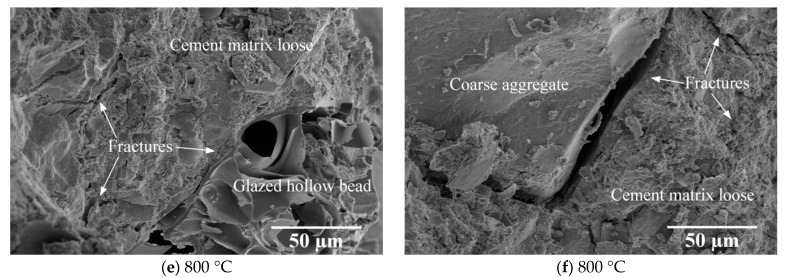
Micro-structures of the interfacial transition zone, exposed to (**a**) 20 °C, (**b**) 200 °C, (**c**) 400 °C, (**d**) 600 °C, (**e**) 800 °C and (**f**) 800 °C.

Figure 5 displays the microstructure of the interfacial zone after exposure to different temperatures. It can be observed that some cement slurry enters into the surface pores of the GHB, forming a tight two-phase mechanical meshing structure at room temperature (Figure 5a). Furthermore, the loss of C-S-H gel adsorption water promotes the cement hydration reaction [28], illustrating the aggregate bond better with the cement matrix after exposure to 200 °C compared to room temperature (Figure 5a,b).

For the temperature above 200 °C, the weight and compressive strength decrease, and the pace of decline is prone to becoming faster as the temperature increases. When the temperature reaches 400 °C, interlayer C-S-H water and a portion of the chemically bonded water from the C-S-H start to evaporate [29]. With the loss of moisture, several tiny cracks can be observed at the interface zone (Figure 5c), indicating their microstructure is not as dense. Moreover, the formation of fractures and pores significantly weakens the mechanical properties.

The compressive strength decreases significantly within 600–800 °C since a further increase in temperature of GHBC to 600 °C causes the decomposition of calcium hydroxide and causes more moisture to evaporate (Figure 5d) [28]. After exposure to 800 °C, the loss of weight is about 10% and the residual compressive strength is only about 30% compared to its strength at room temperature. This behaviour is expected since the C-S-H starts to completely decompose when the temperature reaches around 800 °C [26]. Furthermore, the main component of limestone is CaCO_3_, which decomposes into CaO and CO_2_ [30], resulting in a significant mass reduction and a broken shape with wider cracks between the aggregate and the matrix (Figure 5e,f).

#### 3.1.2. Failure Mode

The failure modes of GHBC specimens, exposed to different temperatures, are presented in Table 4. It can be seen that the failure mode after exposure to different temperatures shows certain characteristics. Two roughly parallel macro-cracks are observed on the failure surface running through the entire specimen at room temperature, showing the characteristics of tensile failure. However, in the specimen subjected to 200 °C, the macro-crack is inclined to a certain angle. The inclination angle of the macro-crack increases with rising temperatures compared to the vertical axis, illustrating the characteristics of shear failure. Multiple cracks connecting to each other are identified and the fractures penetrate more on the surface when the temperature exceeds 600 °C. This is in agreement with previous observations reported by Chen et al. [31].

From the sketch in Table 4, it can be concluded that the failure mode evolves from a single inclined shear plane to a multi-directional inclined shear plane. This is because the internal tiny cracks, induced by high temperature, expand and extend under the high stress. As the temperature rises, the perturbation friction between the shear planes increases, which results in more broken particles emerging on the fracture surface. When the temperature is above 600 °C, the two ends of the specimen are crushed with blocky spalling on the surface. This is in accordance with the drastic deterioration of the microstructure following exposure to 600 °C.

### 3.2. Creep Behaviour

#### 3.2.1. Creep Curves

**Figure 6:**
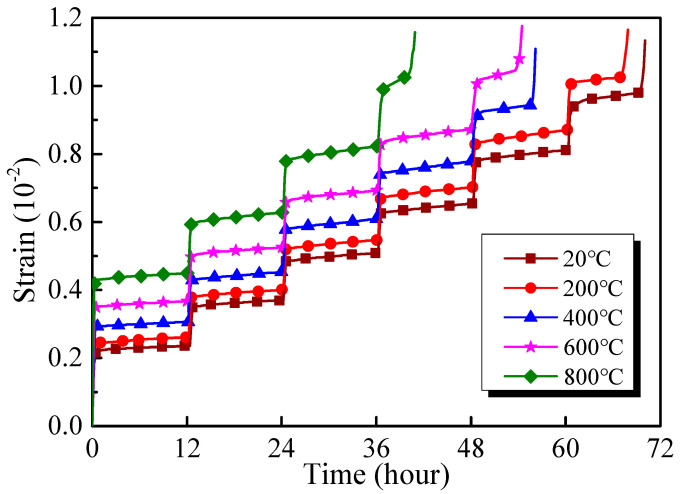
Multistage compression creep curves of GHBC exposed to different temperatures.

The results of creep tests are demonstrated in Figure 6 in the form of strain-time curves. It can be seen that the creep curves of the GHBC specimens after exposure to different temperatures present a ladder form rising under the step load. The creep curve increases abruptly at the moment of loading due to the transient deformation occurrence. When the stress remains constant, the strain increases at a low rate, while the creep curve grows slowly. At a low stress level, the deformation is dominated by the transient strain [32]. As the stress increases, the creep deformation increases, the slope of the creep curve increases, and the creep rate is prone to stabilise, which is characterised by two stages of primary creep and steady-state creep. Under the last stress level, the creep strain response increases exponentially, resulting in the failure of the specimen, which can be divided into three stages including primary creep, steady-state creep, and acceleration creep [33].

Furthermore, the influence of exposure to different temperatures on the concrete creep duration varies. The results indicate that the specimen at room temperature undergoes six stages until its failure, while, after 800 °C, the specimen undergoes four stages. Indeed, the time to failure is reduced from 70.54 h to 40.9 h. It is evident that the rising temperature promotes crack propagation and increases the internal damage, as well as accelerating the concrete failure process.

**Figure 7:**
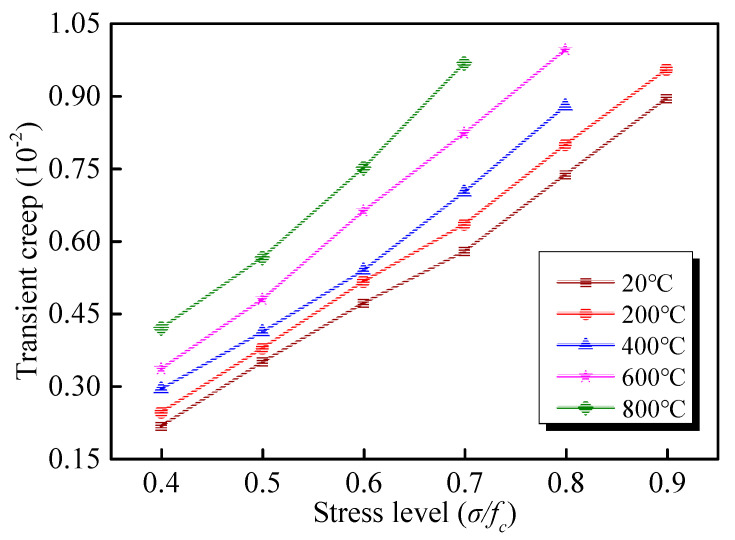
Relationship between the stress level and the transient strain of GHBC, exposed to different temperatures.

The relationships between the transient strain and stress level are demonstrated in Figure 7. As shown, a good correlation exists between the transient strain and stress level, which expresses that the transient strain increases rapidly with the increase of stress level. Under the first stress level (*σ*/*f*_c_ = 0.4), the transient strain is 2.16 × 10^−3^ at the room temperature of 20 °C. The transient strains of the GHBC at 200 °C, 400 °C, 600 °C, and 800 °C are more than that of at room temperature, and the increase percentages are 13.43%, 35.65%, 55.09%, and 93.98%, respectively. Indeed, the increasing temperature weakens the resistance to transient deformation.

**Figure 8:**
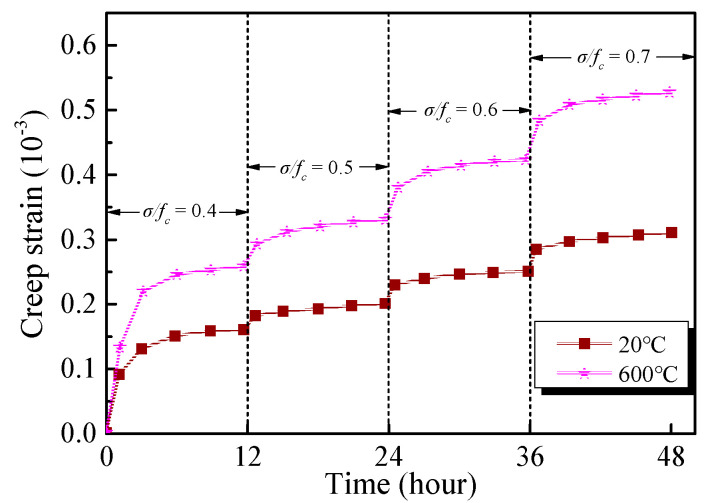
Axial creep strains of GHBC exposed to different temperatures.

Figure 8 presents the creep strain under the stress level between 0.4 and 0.7, exposed to room temperature and 600 °C, respectively. When the stress level is 0.4, the creep strain of the specimen at room temperature is 0.17 × 10^−3^, while that of the specimen after exposure to 600 °C is 0.24 × 10^−3^, i.e., an increase of 41.18%. When the stress level increases to 0.7, the creep strain of the specimen at room temperature is 0.31 × 10^−3^, while, after exposure to 600 °C, the creep strain increases to 0.54 × 10^−3^, showing a 74.19% increase. The above comparison shows that the effect of stress level on the creep behaviour of GHBC is related to the temperature. The influence of temperature on creep deformation under high stress is more significant, given that the high stress aggravates the deterioration of GHBC. Under long-term stress, the cracks and pores are supposed to have sufficient time to expand. Besides, the slip distance between the particles increases, and the edges of coarse particles are crushed, hence the weak particles break, which eventually results in a reduction in creep resistance [34].

#### 3.2.2. Transient Deformation Modulus

The ratio of each stress and its corresponding axial transient strain is defined as the transient deformation modulus *E*_0_, which can be expressed, as follows:

(1) $$E_{0}=\sigma /\varepsilon_{0}$$

**Figure 9:**
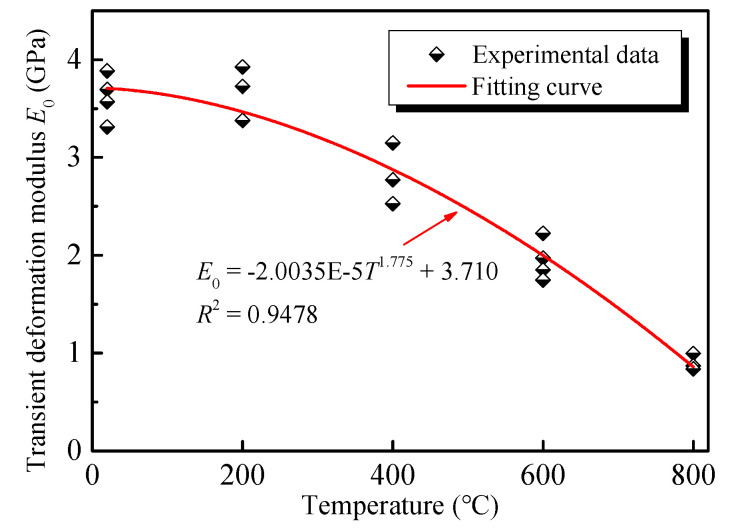
Transient deformation modulus of GHBC after exposure to different temperatures.

The transient deformation moduli *E*_0_ of GHBC exposed to various temperatures under different stress levels are presented in Figure 9. It can be observed the *E*_0_ of the GHBC specimen fluctuates within a certain range, under various stress levels. Therefore, the average value ${\bar{E}}_{0}$ is used to explore the effect of temperature on the transient deformation modulus of the GHBC [35]. With reference to the research by Ma et al. [36], we define the decrease of the ${\bar{E}}_{0}$ after different temperatures as the total deterioration degree *S*_n_ and the average decrease of the ${\bar{E}}_{0}$ after each adjacent temperature as the phase deterioration degree (Δ*S*), which can be calculated as follows:

(2) $$S_{T_{i}}=\frac{{\bar{E}}_{0T_{0}}-{\bar{E}}_{0T_{i}}}{{\bar{E}}_{0T_{0}}}\times 100\%$$

(3) $$\Delta S=\frac{S_{T_{i+1}}-S_{T_{i}}}{\left(T_{i+1}-T_{i}\right)/100}$$

where ${\bar{E}}_{0T_{0}}$ is the average transient deformation modulus of GHBC at room temperature, and ${\bar{E}}_{0T_{i}}$ is the average transient deformation modulus of GHBC when heating temperature is *T_i_*. When the heating temperature *T_i_* is 20, 200, 400, 600, and 800 °C, the corresponding *i* is 0, 1, 2, 3, and 4, respectively.

**Figure 10:**
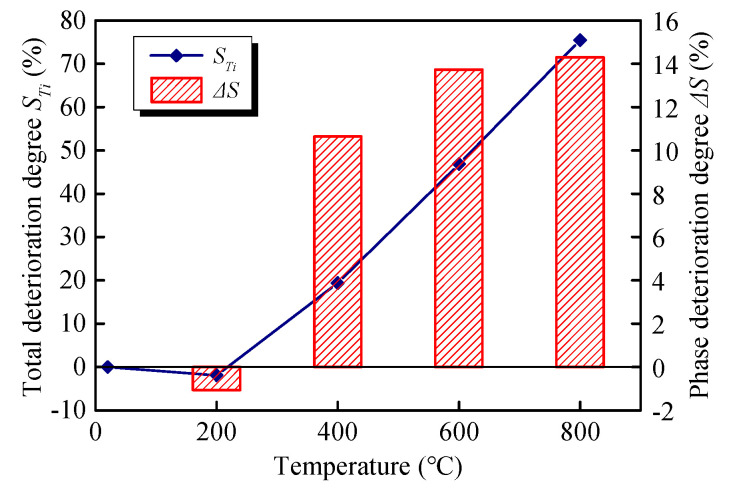
The total deterioration degree and phase deterioration degree of GHBC exposed to different temperatures.

According to the definition in Equations (2) and (3), the trend of the total deterioration degree *S_T_* and the phase deterioration degree Δ*S* of the GHBC specimens exposed to different temperatures are presented in Figure 10.

As presented in Figure 9 and Figure 10, the transient deformation modulus of GHBC after exposure to 200 °C slightly increases with respect to the room temperature, resulting in a negative deterioration degree. This can be attributed to the dense internal structure, which enhances the resistance to transient deformation to some extent. As the temperature increases, the transient deformation modulus of GHBC decreases, while the total deterioration gradually increases. The total deterioration degree of GHBC, exposed to 400 °C, is 19.38%. As the temperature increase, the total deterioration degree increases to 46.85% and 75.44%, respectively. Moreover, the phase deterioration degree increases, indicating that the deterioration of GHBC by high temperature is a cumulative process. As presented, above 600 °C, the total deterioration degree reaches a relatively high value, indicating it triggers the drastic deterioration process of GHBC, which is consistent with the inferior microstructure shown in Figure 5.

#### 3.2.3. Creep Strain Rate

**Figure 11:**
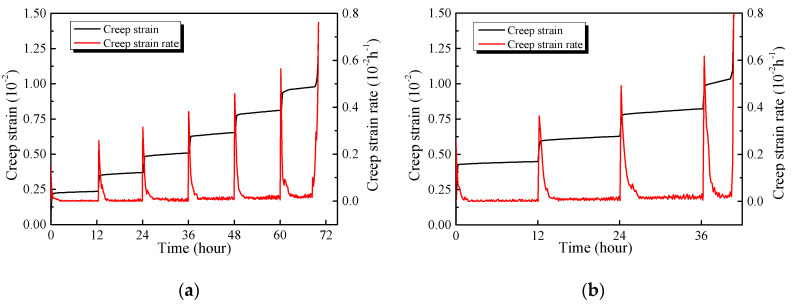
The creep stain rate of GHBC exposed to (**a**) 20 °C and (**b**) 800 °C.

The creep strain rate of the specimen at room temperature and 800 °C under each stress level are demonstrated in Figure 11a,b. The creep rate reaches a relatively high value at the moment of loading (Figure 11a). The latter corresponds to the considerable transient deformation of the specimen and gradual increases of the transient strain rate as the stress level increases.

Once loading is finished, the creep rate decreases sharply. After the decay of the transient rate, the creep rate of the specimen at room temperature remains relatively low, with small fluctuations, when the loading ratio is lower than 0.5. When the stress level fluctuates between 0.6–0.9, the creep rate decreases after the completion of instantaneous creep, and the steady-state creep rate approaches to a constant value. Unlike the creep, the strain is practically constant under a lower stress level during the steady-state creep stage, and the creep strain at the high stress levels continues to grow. At the last stress level, the creep keeps stable for a short period of time after the decay of transient strain, followed by a dramatic increase until the specimen fails.

Furthermore, the fluctuation range of the steady-state creep rate increases significantly as the temperature increases. The variation range of the steady-state creep rate of the GHBC specimen at room temperature is between 0.35 × 10^−5^ and 0.62 × 10^−5^ h^−1^, while that of the range at 800 °C fluctuates between 0.74 × 10^−5^ and 1.88 × 10^−5^ h^−1^. This can be explained by the exposure to the high temperature, accompanied by the formation of cracks and voids. Creep stress accelerates the development of the cracks and the expansion of the internal weakening zone, presenting obvious inhomogeneous characteristics [36].

#### 3.2.4. Accelerated Creep Stage

**Figure 12:**
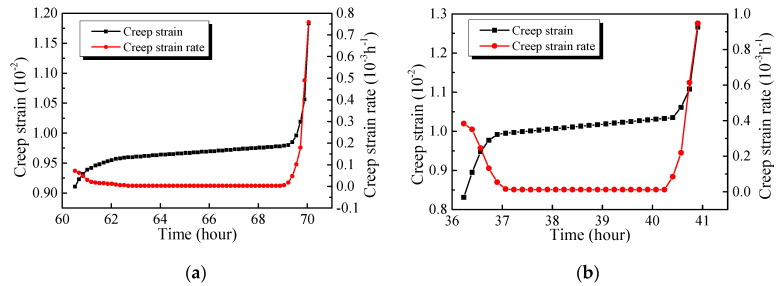
Relationships among strain, rate, and time at the accelerated creep stage, exposed to (**a**) 20 °C and (**b**) 800 °C.

All of the specimens are destroyed at the last stress level. Figure 12a,b represents the curves of creep strain and creep rate under the last stress level for the specimens exposed to room temperature and a high temperature of 800 °C. It can be seen that the creep failures of GHBC specimens, exposed to different target temperatures, follow a similar trend under the last stress level. The specimens develop from the primary creep through the steady-state creep, and finally, the failure occurs at the accelerated creep stage, in which the primary creep and accelerated creep stage show shorter durations, while the accelerated creep stage presents longer durations.

The durations of creep under failure stress are presented in Table 5. It can be seen that the threshold stress of creep failure of the specimen at room temperature is 0.9, while, that of the specimen after exposure to 800 °C is 0.7. Comparing the specimens which failed under the same loading ratio, it can be seen that the higher the temperature, the larger the creep failure strain and the shorter the duration. Hence, the proportions of primary creep and accelerated creep duration to the total creep duration gradually increase, reaching 35.54% and 14.56% after exposure to 800 °C, respectively. Similar results are reported by Li et al. [37].

#### 3.2.5. Critical Stress Level for Creep Failure

To achieve a better understanding of the effect of high temperature on the GHBC creep behaviour, the variable *φ* is defined as the ratio of the creep strain *ε_c_* to the total strain *ε_t_* of the GHBC specimen, exposed to different target temperatures. The calculation formula of *φ* is given, as follows:

(4) $$\varphi =\varepsilon_{c}/\varepsilon_{t}\times 100\%$$

**Figure 13:**
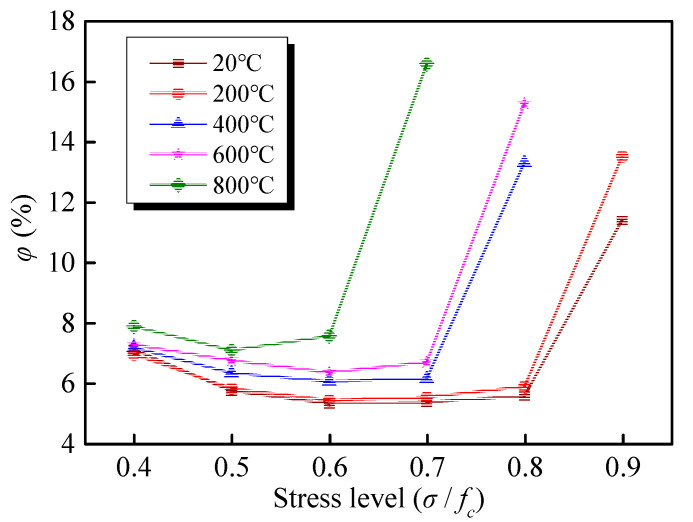
Relationship between the *φ* (ratio of the creep strain to the total strain at each stress level) and the stress level.

According to Equation (4), the trend of *φ* at various stress levels, exposed to high temperature, is presented in Figure 13. It can be discovered that the *φ* decreases under the low stress levels, but when the stress level reaches the critical value, the *φ* increases rapidly with the increase of the stress level. For the specimen at room temperature, the *φ* exhibits a small decrease from 6.89% to 5.80% when the stress level increases from 0.4 to 0.5. When the stress increase to 0.8, the *φ* slightly increases, reaching 5.91%; however, the value of *φ* reaches to 12.30% under the last stress level (*σ/f_c_ =* 0.9). A clear correlation is identified between the *φ* and the stress level. After exposure to >800 °C, although four data points are available, the *φ* first decreases, followed by an increase, which is consistent with the trend of other curves after exposure to different target temperatures.

As presented in Figure 13, each curve has a critical point, dividing the curve into the decreasing and increasing parts. Before the critical point, GHBC exhibits linear creep, at which a slight increase occurs in the creep strain *ε_c_* as well as a large increase in transient strain *ε*_0_, resulting in decreases of the *φ* as the stress level increases. However, after the critical point, GHBC exhibits nonlinear creep, at which the creep strain increases significantly; hence, the *φ* increases as a power function with the stress level. The critical point is 0.7*f_c_* at room temperature and 200 °C, compared to 0.5*f_c_* for the critical point at 800 °C. The critical point moves forward with the increase of temperature, indicating that the GHBC is more prone to instability after exposure to high temperatures. Based on Yu et al. [38], the creep strain of red sandstone specimens can be ignored when the stress level is below 50% of the peak strength. However, the linear creep under the stress below the critical stress level is negligible, while the nonlinear creep occurs when the stress level exceeds the critical point, leading to the creep failure [39].

Results, demonstrated in Figure 13 suggest that this relationship can be served as a design criterion for determining the critical stress level of creep failure via a multistage creep test. The results show that the creep of the GHBC that suffered from high temperature is negligible when the long-term stress is below 50% of the peak strength. Once the stress exceeds the point, the GHBC exhibits nonlinear creep behaviour, and the creep rate is determined by the stress level and temperature condition.

### 3.3. Burgers Model

**Figure 14:**
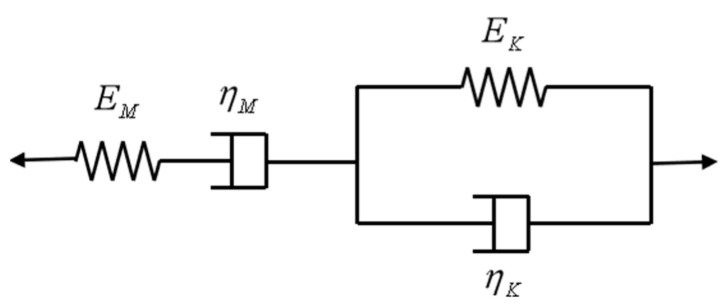
The creep model of Burgers.

The creep curves in Figure 6 demonstrate that the GHBC specimens experience a transient and steady-state creep stage at each stress level. Furthermore, the creep strain rate increases at first, followed by a decrease, until reaching a constant value. Furthermore, the creep behaviour of concrete is usually described by the Burgers model, consisting of the Kelvin model and Maxwell model in series, as shown in Figure 14. The creep equation of the model is as follows:

(5) $$\varepsilon \left(t\right)=\frac{\sigma }{E_{M}}+\frac{\sigma }{\eta_{M}}t+\frac{\sigma }{E_{K}}\left(1-e^{-\frac{E_{K}}{\eta_{K}}t}\right)$$

where, *ε*(*t*) is the strain evaluated at a certain time instant *t*, *σ* is the stress, *E_M_* is the elastic modulus, *E_K_* is the viscoelastic modulus, and *η_M_* and *η_K_* are the viscosity coefficients. The first term in the model represents the transient or the elastic strain, which is independent of time. The second term represents the creep strain, which is time related. Finally, the third term represents the primary creep with a decreasing creep rate [40]. Therefore, the Burgers equation is selected to simulate the GHBC creep behaviour.

**Figure 15:**
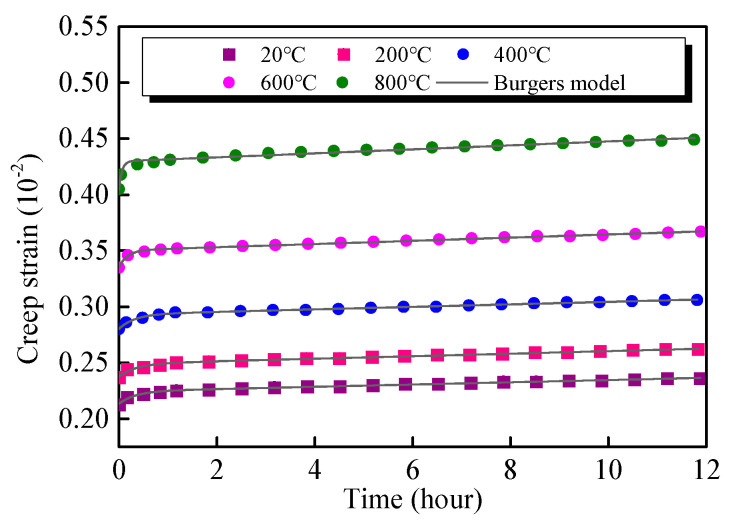
The creep strain of GHBC after exposure to different temperatures for experimental data and theoretical curves under the first stress levels (*σ*/*f*_c_ = 0.4).

Taking the creep test results under the first stress level (*σ/f_c_ =* 0.4) of the specimens exposed to different temperatures as examples, the rheological parameters of *E*_0_, *E*_1_, *η*_1_, and *η*_2_ are identified according to the test data. The creep test data and theoretical curve are plotted and presented in Figure 15. It can be observed that the predicted data agrees well with the experimental data. Table 6 represents the parameters of the Burgers creep equation after exposure to various target temperatures. The values of coefficient of determination *R^2^* are higher than 0.98, indicating that the Burgers model can well reflect the primary creep and steady-state creep behaviour of the GHBC after exposure to high temperature.

The results, provided in Table 6, reveal the transient elastic strain increases and the *E_M_* decreases to some extent as the temperature increases. From Equation (5), the viscosity coefficient *η_M_* is inversely proportional to the creep strain rate at the steady-state creep stage [32]. The creep rate increases when a higher temperature is targeted, resulting in a reduction in the *η_M_*. In addition, the *E_K_* and *η_K_* decrease with increasing temperatures. They exhibit a considerable descending trend above 600 °C, indicating that the ability to resist the elastic deformation and viscous flow decreases sharply after exposure to 600 °C [41,42].

**Figure 16:**
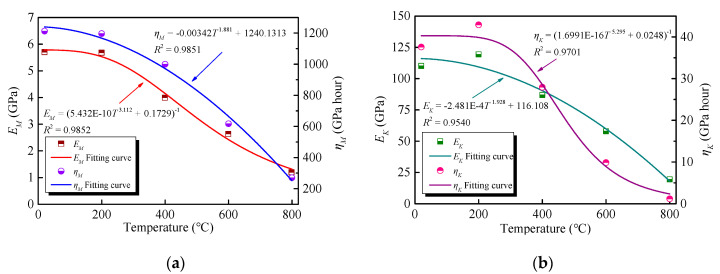
The effect of temperature on (**a**) *E_M_* and *η_M_* (**b**) *E_K_* and *η_K_*.

As mentioned above, the parameters of the Burgers model (i.e., *E_M_*, *η_M_*, *E_K_*, and *η_K_*), presented in Table 6, vary with the temperature. It is essential to determine the parameters of the model under different temperatures to validate its applicability [43,44]. Therefore, data regression analyses are undertaken on the parameters, presented in Table 6. The results, plotted in Figure 16a,b, demonstrate the curves coincide well with the experimental data and the differences are less than 5%. The fitting formulas are as follows:

(6) *E_M_* = (5.432 × 10^−^^10^*T*^3.112^ + 0.1729)^−1^*R*^2^ = 0.9852

(7) *η_M_* = −0.00342*T*^1.881^ + 1240.1313 *R*^2^ = 0.9851

(8) *E_K_* = −2.481 × 10^−^^4^*T*^1.928^ + 116.108 *R*^2^ = 0.9540

(9) *η_K_* = (1.6991 × 10^−^^16^*T*^5.295^ + 0.0248)^−1^*R*^2^ = 0.9701

According to Equations (6)–(9), the *E_M_*, *η_M_*, *E_K_*, and *η_K_* parameters can be calculated to obtain the Burgers model equation, considering high-temperature damage. Once the target temperature and the stress of the GHBC are determined, the creep behaviour under high temperature, predicted by the parameters of Burgers model, can provide the reference for the fire resistance design.

## 4. Conclusions

In this study, a series of uniaxial compression tests and multistage creep tests were carried out to study the influences of different target temperatures on the GHBC. The mechanisms of high temperature on the GHBC were analysed from both perspectives of the failure mode and the internal defects. The main findings of our study are as follows:

(1) The weight and compressive strength of the GHBC decrease as the temperature increases. Moreover, the loss of weight and strength after exposure to 800 °C reach 9.67% and 69.84%, respectively. The failure mode shows the transformation from tensile failure to shear failure as the temperature increases.
(2) The creep strain and creep rate increase with the increase in the temperature and the stress level, while the creep failure threshold stress and creep duration are reduced significantly. The higher the temperature, the more sensitive the stress is to the creep. The creep of the GHBC exhibits a considerable increase above 600 °C and the creep under the same loading ratio at 600 °C increases by 74.19% compared to the creep of the sample at room temperature.
(3) The transient deformation modulus of the GHBC decreases as a power function with increasing temperature, while the deterioration degree increases. The total deterioration degree reaches a relatively high value at 600 °C, indicating it triggers the drastic deterioration process of the GHBC.
(4) The ratio of creep strain to total strain decreases at first, followed by an increase. The inflection point can be considered as the critical stress level of creep failure, which decreases with the increase of temperature.

Finally, the parameters of the Burgers model are identified based on the experimental results. The theoretical curve of the model shows a satisfactory agreement with the creep test data at the primary creep and steady-state creep stages, which could potentially be applied in the fire resistance designs of the GHBC structures.

## Figures and Tables

**Table 1:** The chemical composition of the binder material.

Composition		SiO_2_	Al_2_O_3_	Fe_2_O_3_	CaO	MgO	Na_2_O	SO_3_	Ignition Loss
Content (%)	Cement	22.60	5.03	4.38	63.11	1.46	−	2.24	1.18
	Fly ash	53.26	34.72	4.07	2.47	0.39	1.90	−	4.07

**Table 2:** Performance indicators of glazed hollow bead.

Particle Size/mm	Bulk Density/(kg m^−3^)	Apparent Density/(kg m^−3^)	Cylinder Compressive Strength/MPa	Thermal Conductivity/(W (m K)^−1^)	Refractoriness/°C	Volume Loss Rate at 1 MPa/%
0.5–1.5	80–120	80–130	≥150	0.032–0.045	1280–1360	38–46

**Table 3:** Mix proportions of glazed hollow bead insulation concrete.

Cementing Material		Limestone	Fine Aggregate		Water	Water Reducer	Water Cement Ratio
Cement	Fly Ash		Sand	Glazed Hollow Bead			
421	47	856	571	100	168.48	4.68	0.36

**Table 4:** Failure modes of GHBC specimens exposed to different temperatures.

Legend	Temperature/°C				
	20	200	400	600	800
Failure pattern	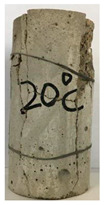	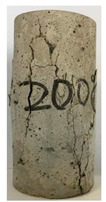	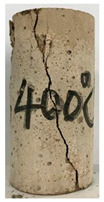	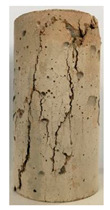	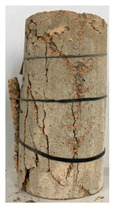
Sketch	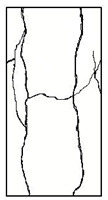	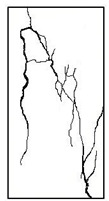	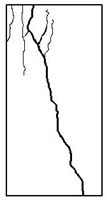	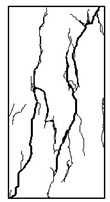	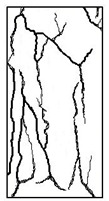

**Table 5:** Durations of creep for GHBC under failure stress.

Temperature/°C	Failure Stress/(*σ*/*f_c_)*	Total Duration/h	Primary Creep Stage		Steady-State Creep Stage		Accelerated Creep Stage	
			Primary Creep Stage Duration/h	Proportion of Total Duration/%	Steady-State Creep Stage Duration/h	Proportion of Total Duration/%	Accelerated Creep Stage Duration/h	Proportion of Total Duration/%
20	0.9	10.54	1.71	16.22	7.82	74.18	1.01	9.58
200	0.9	7.50	1.67	22.27	4.97	66.27	0.86	11.47
400	0.8	7.83	1.34	17.11	5.67	72.38	0.82	10.47
600	0.8	6.17	1.68	27.23	3.70	59.97	0.79	12.80
800	0.7	4.67	1.66	35.54	2.33	49.88	0.68	14.56

**Table 6:** The simulated parameters of the Burgers model.

Temperature/°C	*σ*/MPa	*E_M_*/GPa	*η_M_*/GPa	*E_K_*/GPa	*η_K_*/GPa	*R* ^2^
20	12.2	5.703	1212.181	110.064	37.591	0.9936
200	13.5	5.680	1196.639	119.347	42.899	0.9899
400	11.2	3.990	998.948	86.853	27.934	0.9952
600	8.8	2.628	616.968	57.972	9.866	0.9960
800	4.8	1.185	269.986	19.374	1.193	0.9831
